# Basic Conditional Reasoning: How Children Mimic Counterfactual Reasoning

**DOI:** 10.1007/s11225-013-9510-7

**Published:** 2014-08-01

**Authors:** Brian Leahy, Eva Rafetseder, Josef Perner

**Affiliations:** Department of Linguistics, Universiẗat Konstanz, Fach D-185, 78457, Konstanz, Germany, brian.leahy@uni-konstanz.de; Department of Psychology, University of Salzburg, University of Konstanz, Fach D-9, 78457, Konstanz, Germany, eva.rafetseder@sbg.ac.at; Department of Psychology and Centre for Neurocognitive Research, University of Salzburg, Hellbrunnerstrasse 34, 5020, Salzburg, Austria, josef.perner@sbg.ac.at

**Keywords:** Counterfactual Reasoning, Basic Conditional Reasoning, Counterfactuals, Possible worlds semantics, Generic reasoning

## Abstract

Children approach counterfactual questions about stories with a reasoning strategy that falls short of adults’ Counterfactual Reasoning (CFR). It was dubbed “Basic Conditional Reasoning” (BCR) in Rafetseder et al. (Child Dev 81(1):376-389, 2010). In this paper we provide a characterisation of the differences between BCR and CFR using a distinction between permanent and nonpermanent features of stories and Lewis/Stalnaker counterfactual logic. The critical difference pertains to how consistency between a story and a conditional antecedent incompatible with a nonpermanent feature of the story is achieved. Basic conditional reasoners simply drop all nonpermanent features of the story. Counterfactual reasoners preserve as much of the story as possible while accommodating the antecedent.

## Introduction and Overview

In a study described in [9], children observed a puppet named Carol making dirty footprints on a clean floor. They were then asked, “What if Carol had taken her dirty shoes off-would the floor be clean or dirty?” 75% of 3-year-olds and 87% of 4-year-olds answered that the floor would be clean. It was concluded that 3- and 4-year-olds are typically capable of counterfactual reasoning. However, consider this story, played out with dolls with narration by an experimenter, and question (I) subsequently asked about the story:

Marie is walking to the swimming pool in her swimsuit. On her way to the pool she is caught in a rainstorm and gets soaked. Then she gets to the pool and jumps in. Now she is all wet.

### (I) If it hadn’t rained, would Marie be wet or dry?

[18] shows that children perform poorly on this and similar tasks. At age 5, children answered “wet” only about 18% of the time. Even at age 10, only about half of counterfactual questions like (I) are answered correctly. 12-year-olds answered “wet” 88% of the time. In this paper we assume that ‘wet’, the near-universal adult answer, is correct.

This task simplifies an earlier task that had generated similar results [17]. Children were shown the following story with a dollhouse and puppets.

There is a dollhouse with two bedrooms and a kitchen. One bedroom belongs to a little girl; the other to her older brother. In the kitchen are two shelves, a top shelf and a bottom shelf. The little girl can reach the bottom shelf but is too short to reach the top shelf. Her older brother can reach both shelves. The children’s mother sometimes brings home sweets. Sometimes she puts them on the top shelf, and sometimes on the bottom shelf. When she puts them on the bottom shelf, then whichever child comes looking for sweets will find them and take them back to their room. But when she puts them on the top shelf, only the older brother can reach them. So if the boy comes, the sweets will end up in his room. If the little girl comes, they will stay on the top shelf.

Several control questions were then asked to ensure that subjects understood the structure of the situation. Then conditional questions about the situation were posed. Children performed very well when asked conditional questions about the situation whose antecedents described possible future events. For example, if the experimenter continued by showing the mother coming home and putting sweets on the top shelf, even children whose ages ranged from two years and eleven months to five years and nine months most often correctly answered the question, “If the little girl comes looking for sweets, what will happen to them? Where will the sweets be?” by saying that the sweets would remain on the top shelf. But when they were shown the mother coming home and putting sweets on the top shelf, and the older brother retrieving them to his bedroom, the same children gave incorrect answers to the counterfactual question (II) 94% of the time.

### (II) If not the older boy but rather the little girl had come, where would the sweets be?

39 of 66 incorrect responses indicated the little girl’s room.

This data was explained by drawing a distinction between Counterfactual Reasoning (CFR) and Basic Conditional Reasoning (BCR), where the ability for CFR typically emerges later than the ability for BCR.^1^ In Harris’s task [9], both strategies yield the same results; the tasks in [17] and [18] were designed so that BCR and CFR would yield different answers, allowing the experimenters to distinguish true counterfactual reasoners (CF reasoners) from basic conditional reasoners (BC reasoners).

The purpose of this paper is to make fully precise what CFR and BCR are by describing in detail what people who answer (I) and (II) via BCR do and contrasting that with what people who answer (I) and (II) via CFR do. We argue that CF reasoners take all information provided by the experimenter into account. BC reasoners systematically fail to take certain kinds of information from the story into account when faced with a counterfactual question whose antecedent conflicts with that information. This paper will describe in detail the kinds of information that BC reasoners exclude and show how this results in a reasoning process that requires only a proper subset of the abilities required for CFR. We use the tools of counterfactual logic (especially [13]) to clarify when and how these systematic differences result in different answers to counterfactual questions.

We do not aim to describe the psychological reality of CFR and BCR as reasoning processes. Our logical characterizations of CFR and BCR make idealizations that are psychologically implausible. The implementation of these logical characterisations is an open issue of psychological significance. Nor do we wish to commit ourselves to possible worlds logic. Our commitments in this respect are briefly discussed in the conclusion.

We will not say in general what we take a counterfactual question to be, as there is as yet no satisfactory theoretical delineation of counterfactual conditionals (see [2,3,14] for discussions of the challenges). It must for now suffice that (I) and (II) are examples of counterfactual questions.

The data we wish to explain derives from experiments where an experimenter provides a test subject with information both visually (using puppets and props) and verbally, and then asks a conditional question whose antecedent contradicts a nonpermanent feature from the information provided. We will treat the information provided as a set of propositions, where a proposition is a set of possible worlds. The intersection of a set of propositions—again a set of possible worlds—is the set of worlds where every proposition in the set is true.

Section 1 of this paper distinguishes between permanent and nonpermanent features, which allows us to describe the propositions that BC reasoners fail to take into account when answering counterfactual questions. Section 2 describes the abilities that both CF and BC reasoners share. This includes (Section 2.1) the ability to identify the best worlds in the intersection of some set of propositions according to a given order and (Section 2.2) the ability to generate the answer to a counterfactual question on the basis of a given set of antecedent worlds. Section 3 describes how the set of antecedent worlds used to answer a counterfactual question is determined. Section 4 makes clear that the abilities required for BCR are a proper subset of the abilities required for CFR. The fact that CF reasoners take more of the propositions provided by the experimenter into account than do BC reasoners when faced with a counterfactual question complicates the process of establishing the set of worlds relevant to answering the question. Section 5 introduces a hypothesis about generic reasoning that can answer an open question in the data reported in [17]. Section 6 concludes.

## 1. What BC Reasoners Neglect

When answering counterfactual questions, CF reasoners take all information provided by the experimenter into account. BC reasoners faced with a conditional question whose antecedent is inconsistent with a nonpermanent feature of the story provided by the experimenter do not take into account any propositions about nonpermanent features of the story provided prior to the asking of the question. They do take permanent features of the story into account. The next paragraphs clarify the terms of this claim.

First a caveat. We use actual world knowledge when we interpret stories. If we hear a story that begins “John spotted Ann from across the room,” we will assume, until the story gives us reason to suspect otherwise, that John is a man and that Ann is a woman, that John has eyes and that those are what he used to spot Ann, and so on. Thus the features of a story are not determined only by the propositions expressed by the sentences in the story. Interpreters import assumptions from the real world [19]. These assumptions need to be treated as features of the story, as it is taken by the interpreter.

Now, what are the permanent features of a story, and what are the nonpermanent features? We cannot at this point present a detailed theory. Instead we provide a two-step test for permanence, and describe how our theory of the difference between BCR and CFR predicts BC reasoners will behave on these tests. Some limitations of this test are described in Section 5.

Given a feature *f* of the story, one first asks a subject, “Can *f* be different in a different episode of this story?” If the subject answers negatively, then *f* is a permanent feature of the story for that subject. A negative response at the first stage is sufficient, but not necessary, for permanence. If an affirmative answer is given, a followup question is asked: “Is *f* normally the case in the story?” A negative answer to this question and an affirmative answer to the first are individually necessary and jointly sufficient for being a nonpermanent feature for that subject. An affirmative answer to the second question and an affirmative answer to the first question is sufficient for being a permanent feature for that subject.

Neither test is fully adequate on its own. For example, the question, “Is the older brother normally tall” is an odd question, and so may not be reliably affirmed or denied by either BC or CF reasoners. (For this reason the question “Can the boy be short in a different episode of this story” should be posed first: a negative answer renders the odd question unnecessary.) Since an affirmative answer to the first question about a feature is consistent with that feature being permanent or nonpermanent, the followup second test is also required.

We now briefly illustrate by describing our predictions regarding how BC reasoners will answer these questions about selected features from the stories described above.

If a reasoner treats the fact that it was raining in the swimming pool story as a nonpermanent feature, we predict that they will answer affirmatively to, “Can it be dry out in a different episode of this story?” and negatively to, “Is it normally raining in the story?”. This latter must be qualified: it must be clear that the reference class for the quantifier “normally” is not just the events of the story as told, but also includes broader features of the world in which the story occurs, imported from actual world knowledge.

We are committed to the claim that BC reasoners take it as a permanent feature of the sweets story that when the little girl finds sweets on the bottom shelf, she takes them back to her room. That means that BC reasoners must either respond negatively to the question, “Can it be that the little girl finds sweets and takes them somewhere else in a different episode of this story?” or respond affirmatively to the question, “Does the little girl normally take sweets to her room when she finds them in this story?”.

When BC reasoners encounter a conditional question about a story, they first try to fit the antecedent in with the story. Thus BC reasoners typically answer indicative questions correctly, as their antecedents do not conflict with any features of the story. But when faced with a conditional question whose antecedent contradicts a nonpermanent feature of the story, they generate their answer based only on permanent features of the story. They ignore all nonpermanent features of the story. When BC reasoners cannot fit the antecedent with the story without generating a contradiction, they “reset” the story: all propositions about nonpermanent features are dropped from the set of propositions attended to. It is as though the story is amended; a new episode begins. But the background of permanent features of the story remains in place.

We must note that we do not have data regarding what BC reasoners do when faced with a counterfactual question about a story whose antecedent is inconsistent with a permanent feature of the story. For example, subjects who see the tall boy removing sweets from the top shelf to his room might be asked, “What if the boy had been as short as his sister? Where would the sweets be?”. All existing experiments used questions whose antecedents contradicted propositions about nonpermanent features.

## 2. What Do Reasoners of Both Varieties Do?

### 2.1. Both BCR and CFR Require Identifying Scenario Worlds

We have just described the respects in which the set of propositions taken into account by the BC reasoner faced with a counterfactual question that contradicts a nonpermanent feature of the story differs from the set of propositions that the CF reasoner takes into account. But given a set of propositions, the same operations must be performed.^2^ They must both (a) find the intersection I of the set of propositions, and (b) find the worlds in I that are maximally similar to the actual world @, according to a similarity order $_@_ that fixes, for any pair of worlds, which if either is more similar to @.^3^ The maximally similar worlds in I are the worlds as they might be if each member of I is true; call their set the scenario worlds, S for short.

**Figure 1:**
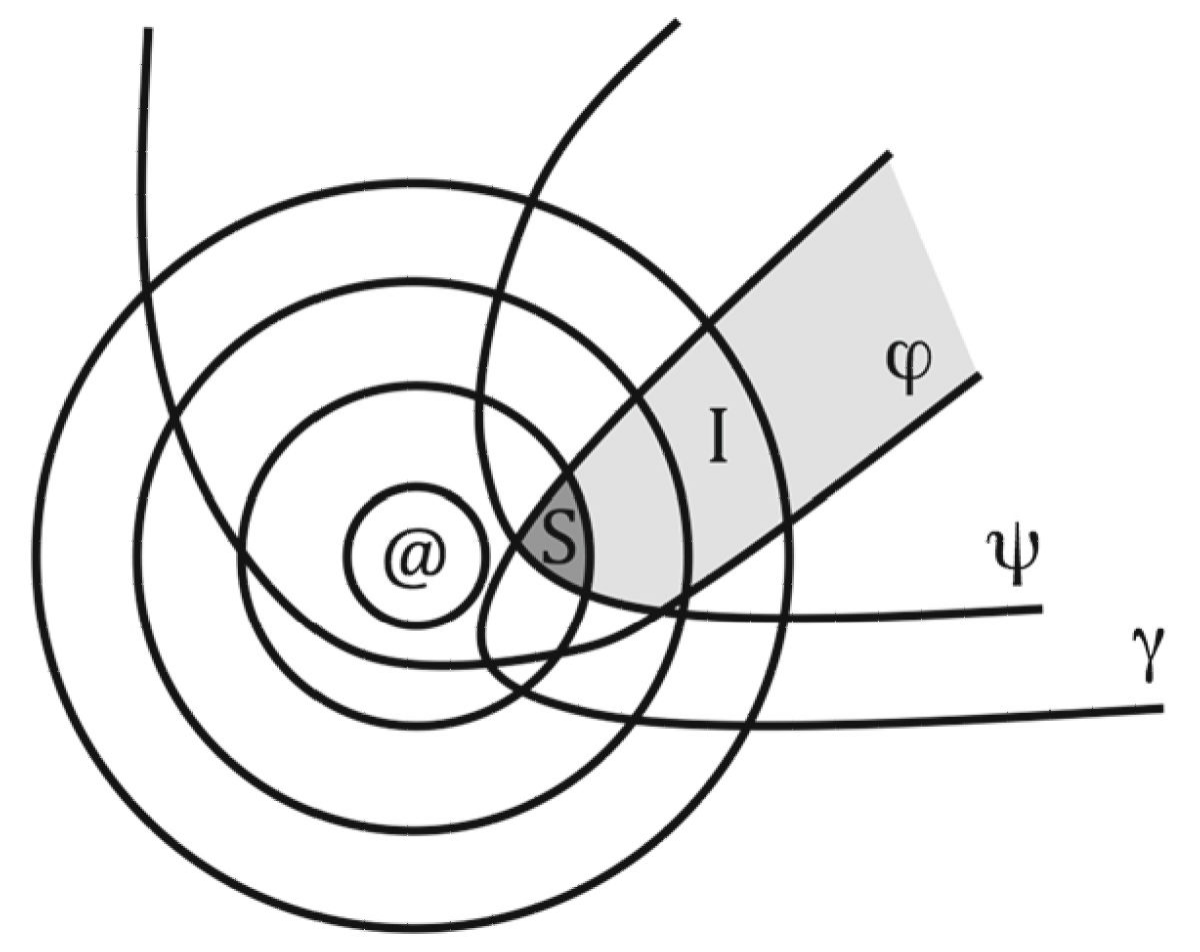
Similarity ordering: a nested set of spheres

Consider Figure 1. Following [13], we represent a similarity ordering of the set of possible worlds relative to @ as a nested set of spheres around @. Any two possible worlds that are equally similar to @ according to $_@_ are members of all the same spheres. The spaces designated *ϕ*, *ψ*, and *γ* are propositions—the sets of all worlds where *ϕ*, *ψ*, and *γ* (respectively) are true. I—the total shaded area of the figure—is the intersection of *ϕ*, *ψ*, and *γ*, i.e., the set of worlds where the conjunction *ϕ*&*ψ*&*γ* is true. S—the darkest shaded area of the figure—is the scenario worlds, the set of best worlds in I according to $_@_.

Members of S should be maximally similar to @ because reasoners should not allow that the story may behave in manners unlike @ unless they are given a reason to think it does by the experimenter.

### 2.2. Question Worlds and Answers to Counterfactual Questions

BC reasoners and CF reasoners also share the ability to generate answers to counterfactual questions “A□→C?” from a set of worlds that we call the question worlds, Q. Briefly, Q is the set of A-worlds that a test subject takes to be the best A-worlds. This is spelled out in the next section, since what A-worlds are taken to be best varies with reasoning strategy. But since the logical features of the relationship between Q and the answer to the question are invariant across reasoning strategies, we describe those features here.

Call the question expressed by the consequent of a counterfactual question the *consequent question*. For example, (I) “If it hadn’t rained, would Marie be wet or dry?” is a counterfactual question; its consequent question is ‘Is Marie wet or dry?’. If the answer to the consequent question is the same at every world in Q, then the answer to the counterfactual question is the same as the answer to the consequent question at any world in Q. We take this to be an application of the standard possible worlds semantics for counterfactual assertions, where a counterfactual A□→C? is true if and only if C is true at every relevant A-world.

If the answer to the consequent question is not the same at every world in Q, this mechanism generates no answer. If forced to generate an answer, we propose that subjects try to find a clear majority, amongst the worlds in Q, that agree on the answer to the consequent question. The answer to the consequent question is the one provided by this clear majority. This is supported by the fact that adults agree on the answers to many counterfactual questions asked “out of the blue,” that is, absent as much supporting context as possible. Asked (I) and similar questions out of the blue, adults robustly agreed that Marie would be dry.^4^ Anecdotal evidence suggests that it is common to reason as follows: “Usually, when it is not raining, people are dry. Assuming Marie is a person, then usually, when it is not raining, Marie is dry. So Marie would be dry.” We presume, then, that most of the closest worlds where it is not raining are worlds where Marie is dry. So, if forced to answer a counterfactual question about the issue, ‘dry’ is a better answer than ‘wet’.

If there is no clear majority among the worlds in Q that agree on the answer to the consequent question, then this process will not generate an answer and some other method must be employed (for example, guessing).

### 3. Q Depends on Propositions Attended To

Section 1 showed how CF reasoners and BC reasoners attend to different propositions when faced with a counterfactual question about a story whose antecedent contradicts nonpermanent features of the story. Section 2 described two abilities that both kinds of reasoners share. This section describes how Q is determined by the propositions attended to, an initial ordering $_@_ , and a counterfactual question.

Given the intersection of a set of propositions I, an order $_@_ , and counterfactual question A□→C?, Q is as follows. If there is a world in I where A is true, Q is the $_@_-best worlds in I where A is true. These will be the A-worlds in S (where S is the set of scenario worlds, the $_@_-best worlds in I) if there are any. In other words, the answer to the counterfactual question is determined by the answer to the consequent question at the worlds most similar to @ where all features of the story are true and the antecedent is true, if there are any such worlds. For if there is a world where I is true and A is true, that is a more relevant world for answering the counterfactual question than is any world where A is true but I is false. And I&A worlds that are more similar to @ should be used as question worlds before I&A worlds that are less similar to @.

**Figure 2:**
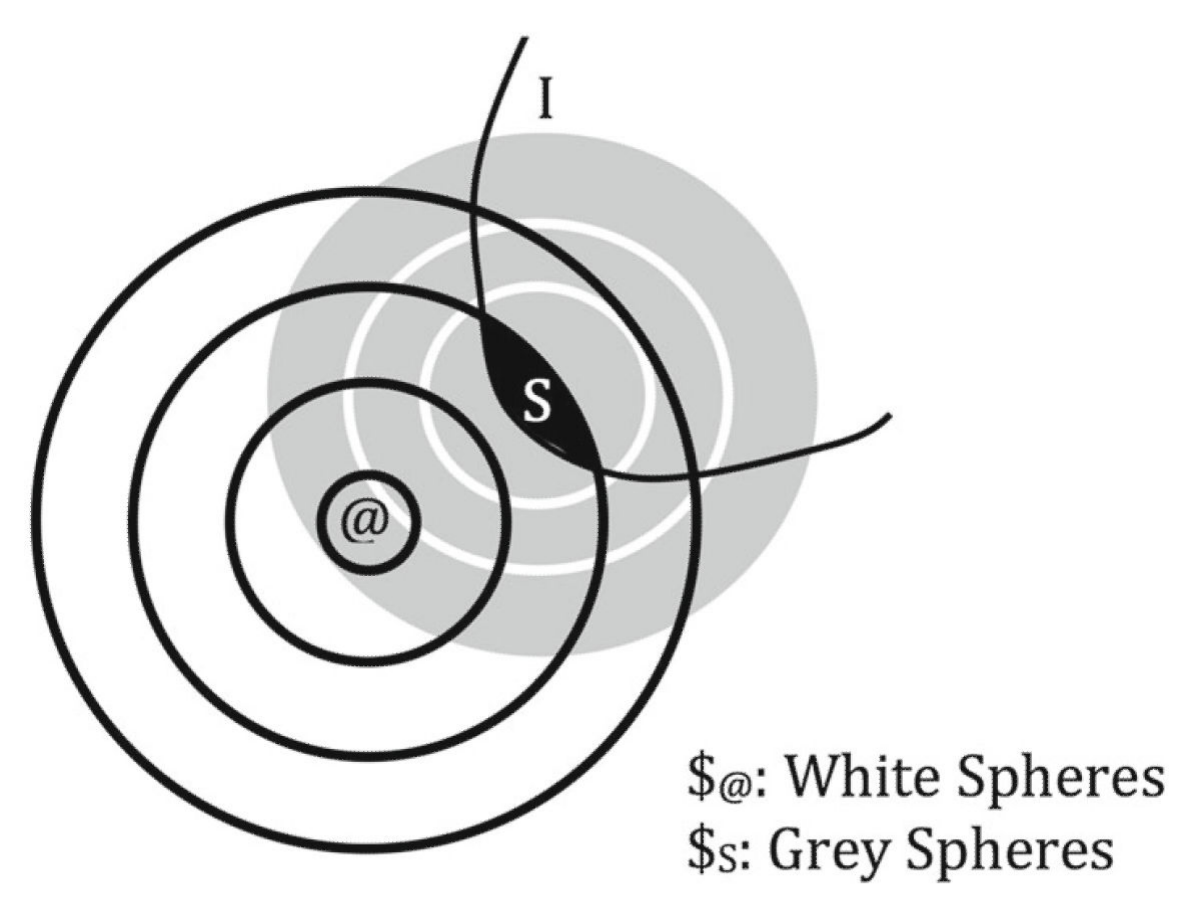
A system of spheres strongly centered on @ and a system of spheres weakly centered on S

**Figure 3:**
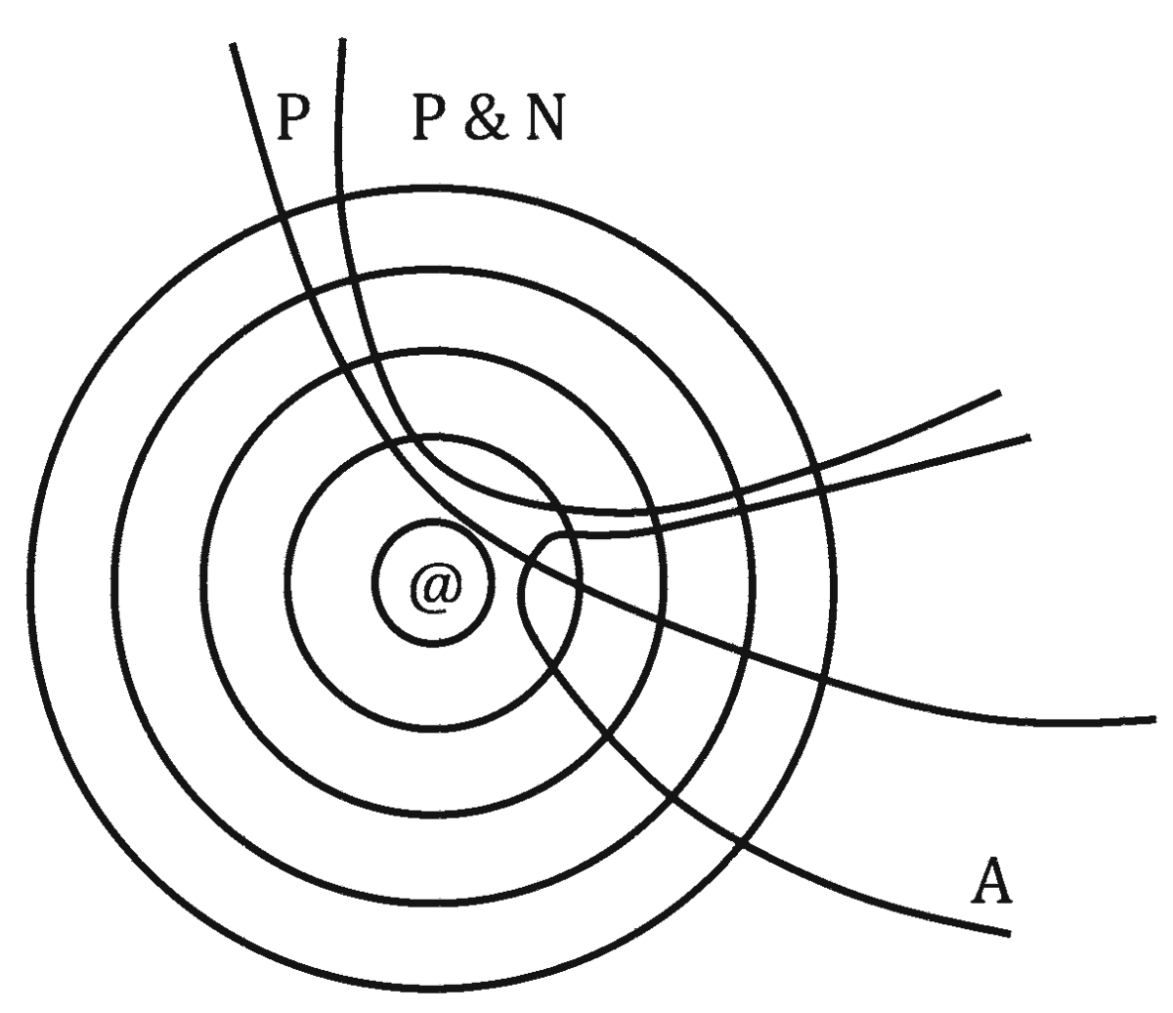
A is consistent with P but inconsistent with (P&N)

**Figure 4:**
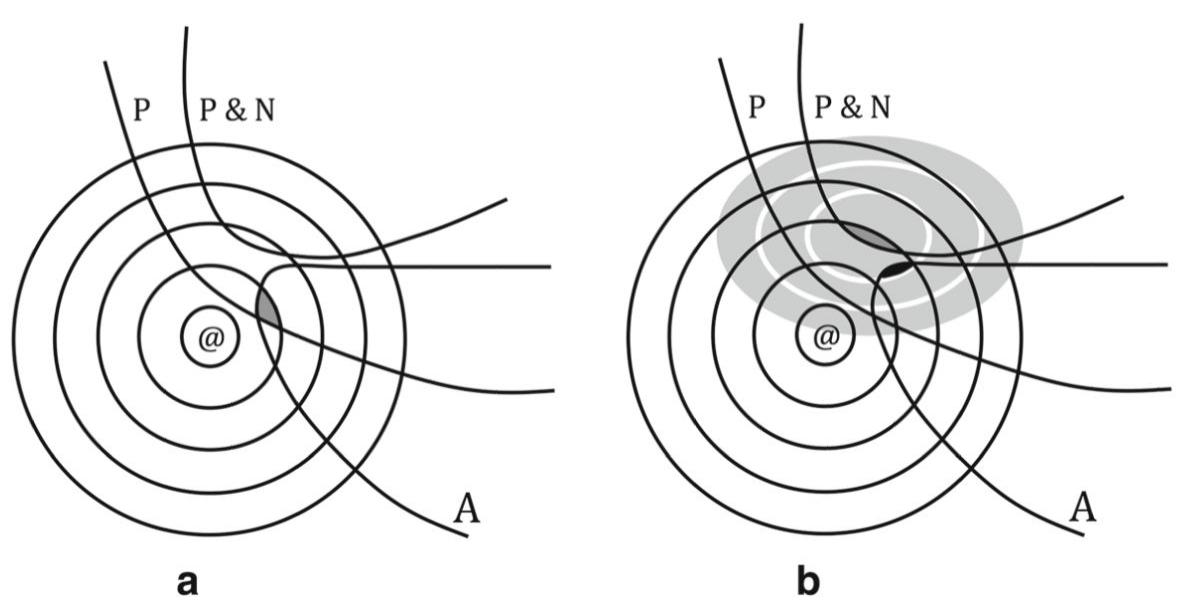
(a) Question worlds for the basic conditional reasoner; (b) question worlds for the counterfactual reasoner

If A is inconsistent with I, reasoners construct a new ordering $_*S*_ that is weakly centered on S.^5^ Then the question worlds are the best worlds according to $_*S*_ where the antecedent of the question is true. These are the story worlds as they would be if the antecedent of the counterfactual question were true. In order to do so they must use an ordering centered on the scenario worlds, not on @. Figure 2 represents the relation between an order $_@_ centered on @, the intersection of a set of propositions I, and a second similarity order $_*S*_ (a formal definition of $_*S*_, a system of spheres weakly centered on a set of worlds S, is provided in footnote 3). The nested white discs represent the ordering $_@_ ; I is the set of worlds where all propositions a test subject takes into account are true. The darkest region is the set S of best worlds in I according to $_@_ . The grey discs are the ordering $_*S*_ of the set of possible worlds that is weakly centered on the members of S. Figures 3 and 4a/b, discussed below, illustrate how the set of question worlds are determined in different ways depending on whether A is consistent with or inconsistent with I.

## 4. Why CFR is Harder than BCR

Why should BCR emerge before CFR? When faced with a counterfactual question about a story whose antecedent contradicts a nonpermanent feature of the story, CF reasoners take into account both permanent and nonpermanent features of the story. BC reasoners presented with the same question only take permanent features of the story into account. So the antecedent of the question is consistent with the features that the BC reasoner takes into account but inconsistent with the features that the CF reasoner takes into account. When the antecedent of a question is consistent with the features a reasoner takes into account, there is no need for that reasoner to construct an order $_*S*_ of the possible worlds centered on the scenario worlds. One answers the question on the basis of the answer to the consequent question at the best antecedent worlds in I according to $_@_ . It is when the antecedent of the conditional is inconsistent with I that reasoners must re-order the set of possible worlds around the scenario worlds in order to find Q.

Consider the sweets story, and question (II) “If not the older boy but rather the little girl had come, where would the sweets be?”. The antecedent of question (II) is “Not the older boy, but rather the little girl came.” This is inconsistent with the set of propositions taken into account by the CF reasoner, which includes the proposition that the older boy and not the little girl came. As a result, in order to generate an answer to the question, the CF reasoner must construct a new system of spheres around her scenario worlds. Just as $_@_ provides an order of the set of possible worlds in terms of their similarity to the actual world, this new system of spheres must provide an order of the set of possible worlds in terms of their similarity to the scenario worlds. Then the reasoner can find the closest worlds to the scenario worlds where the little girl and not the older boy came; those are her question worlds Q. So when the antecedent of the counterfactual question is inconsistent with the set of propositions taken into account, re-ordering the set of possible worlds around the scenario worlds is a necessary task. Otherwise, re-ordering the set of possible worlds around the scenario worlds is unnecessary. This is illustrated in Figure 3, where P is the conjunction of propositions about permanent features of the story; N is the conjunction of propositions about nonpermanent features of the story; and A is the set of worlds where the antecedent of the counterfactual question is true. CFR is more difficult than BCR when A is consistent with P but inconsistent with (P&N).

Another way to express the difference between CFR and BCR is that the CF reasoner has a refined method for restoring consistency to an inconsistent set of premises. The BC reasoner uses a blunt tool: faced with a counterfactual question about a story whose antecedent contradicts a nonpermanent feature of the story, she drops all propositions about nonpermanent features of the story. Then, since the antecedent of the counterfactual question is about a nonpermanent feature of the story, there is no inconsistency between the antecedent and the set of propositions she takes into account. The CF reasoner, faced with a counterfactual question about a story, restores consistency while preserving as much as possible the structure of the story provided by the experimenter.

We further illustrate our hypothesis by showing why BCR and CFR yield different answers to question (I) but why we think they yield the same answers to the question in Harris’s task [9]. First, question (I): If it hadn’t rained, would Marie be wet or dry? The experimenter’s story determines a set of permanent features, including that there is a person named Marie, that Marie is a girl, and so on. There is also a set of nonpermanent features, which includes that it starts to rain while Marie is on her way to the pool; that she gets wet in the rainstorm; that she jumps in the pool and gets wet. When faced with question (I), BC reasoners take the permanent features into account, but not the nonpermanent features. They find the closest worlds to the actual world where all permanent features of the story are true and where it did not rain. Since they do not take into account the fact that it rained, there is such a world. (See Figure 4a, where the shaded region represents the set of best antecedent worlds relative to the set of propositions that the BC reasoner attends to and her initial ordering $_@_ of the set of possible worlds relative to @.) Then, at all such worlds, they check to see whether Marie is wet or dry. Since it is a feature of the actual world that people are usually dry when it is not raining, we presume that in most of these worlds Marie is dry. Therefore the answer generated is that Marie would be dry.

CF reasoners take all members of both sets of propositions into account. As a result there is no world in the intersection of the set of propositions they take into account where the antecedent is true. So they must find the worlds that are maximally similar to the scenario worlds where the antecedent is true. Since these are all worlds where Marie is wet from jumping into the pool, the answer generated is “wet.” (See Figure 4b, where the blackened region in A represents the set of antecedent worlds that are most similar to the scenario worlds according to the order $_*S*_. $_*S*_ is represented as the shaded system of spheres weakly centered on S, which is darkly shaded.)

Consider Harris’ example. There permanent features of the story include that there is a white floor, a person named Carol, etc. Nonpermanent features of the story include that Carol walked across the floor with dirty shoes and that Carol left a trail of footprints. Facing the question, “What if Carol had taken her shoes off—would the floor be clean or dirty?”, BC reasoners find the closest worlds to the actual world where the permanent features of the story are true and where Carol took her shoes off. Since it is a feature of the actual world that floors usually stay clean when people take their dirty shoes off—at least, cleaner than they do when people fail to take their dirty shoes off—we presume that in most of these worlds the floor is (relatively) clean. Thus the answer generated is that the floors would be clean. CF reasoners take the members of both sets of propositions into account. As a result there is no world where all the propositions in those two sets are true and where Carol took off her shoes. So they find the worlds that are maximally similar to the worlds where the propositions in both sets are true and where Carol took her shoes off. Since these are all worlds where the floor stays (relatively) clean, the answer to the question is ‘clean’.

The theory developed in this paper has lead to new empirical questions. What happens when BC reasoners are faced with counterfactual questions whose antecedents are inconsistent with a permanent feature of the story? For example, suppose test subjects in the sweets scenario observe the mother leaving sweets on the top shelf, and the older boy taking sweets back to his room. How would BC reasoners respond to counterfactual questions like, “What if the boy had been as short as his sister? Where would the sweets be?” How BC reasoners deal with such questions is an open empirical question.

## 5. How Things Generally Are

In one version of the sweets story, children saw the mother put sweets on the top shelf and the older boy take them to his room. They were asked question (II): “If not the older boy but rather the little girl had come, where would the sweets be?”. [17] observed that BC reasoners most often answer that the sweets would be in the little girl’s room, while CF reasoners reply that they would be on the top shelf. This is surprising, for while BC reasoners do not take the proposition that the sweets are on the top shelf into account, we do not see why they should assume that the sweets are on the bottom shelf, where the little girl can reach them. Thus an open question is why BC reasoners regularly indicate the little girl’s room, instead of insisting that they need information about where the sweets are or just guessing.

The phenomenon is in fact even broader. While children who see the sweets on the top shelf correctly answer the indicative conditional question, “What if the little girl comes? Where will the sweets be?”, [15] showed that when 6- to 9-year-old children are told that the mother has put the sweets on one of the shelves but are not told which one, they often incorrectly answer the question “What will happen to the sweets if the little girl comes looking?”. 71% (n = 17) said that they would end up in the girl’s room. Only one correctly noted that more information was required.

An explanation for this phenomenon that we find worthy of further investigation has it that the generalization that “the little girl brings sweets to her room” is represented generically. Generic generalizations are known to behave differently from overtly quantified generalizations. While the truth value of generics frequently coincide with the truth values of generalizations overtly quantified by ‘usually’ or ‘always’ (call these “preponderance-” and “universally quantified” generalizations, respectively) they can also come apart. It is true that dogs have four legs, while it is false that all dogs have four legs. It is false that people are right handed, while it is true that most people are right handed. It is true that sperm fertilize eggs while it is false that most sperm fertilize eggs. Furthermore, while the truth value of a generic often coincides with the truth value of its existentially quantified counterpart, their truth values can also come apart. Though some people are left handed, ‘People are left handed’ is not a true generic. Possible worlds semantics for generics are found in, for example, [16].

A number of features play a role in whether a generic claim is accepted as true. [11] makes the case that, for artifacts and social kinds, one of the relevant features is purpose or function. She claims that when it is a function or purpose of members of an artifactual or social kind K to F, then the generic ‘Ks F’ may be true even if no K ever Fs. We adopt the weaker claim that when it is a function or purpose of members of an artifactual or social kind K to F, then the generic ‘Ks F’ may be true even if few Ks ever F.

We may treat the little girl’s visits to the kitchen as a social kind, and it is indeed a purpose of those visits to find sweets so they can be taken to the bedroom. Thus, BC reasoners needn’t assume that the sweets are on the bottom shelf in order to generate the answer that they do in fact produce. They may instead assume that (generically) the little girl takes sweets back to her room, even if they don’t assume that the little girl usually takes sweets back to her room. So the hypothesis that BC reasoners accept this generic could account for the observations. When the information that the sweets are on the top shelf is absent, this generic information may sometimes be relied on. But when information that the sweets are on the top shelf is available, it is not. This hypothesis squares well with a large body of evidence describing the role played by generic reasoning for young reasoners ([4,8,10,12], studies 4A, and 4B) and the interest even 3-year-olds show in generic claims ([12], studies 2A, 2B, and 3). Furthermore, reasoning from this generic may usually be a better strategy than simply guessing, one of the other major options left open for the BC reasoner in this situation.

This hypothesis introduces problems, though, for our two-stage test of nonpermanence from Section 2. At the second stage a question is asked that employs a preponderance quantifier, “normally”. But as noted, the truth values of preponderance quantified sentences can differ from that of the corresponding generic sentences. So there can be a proposition p such that it is normally the case that p, but not generically the case that p. As a result, our test may conclude that p is a permanent feature of a story, though if the generic account is right, p is not treated as a permanent feature of the story by such reasoners. On the other hand, there can be a proposition q such that it is not normally the case that q, but it is generically the case that q. Our test may conclude that q is a nonpermanent feature of a story, despite being attended to by such reasoners.

This yields testable hypotheses. In situations where generics and preponderance quantified sentences yield different results, does the behaviour of BC reasoners agree with the hypothesis that permanent features of stories are those that pass the two-stage normally test, or with the hypothesis that permanent features of stories are those that pass a suitably modified version of the two-stage normally test that employs generics instead of ‘normally’? BC reasoners who have seen the older boy take sweets from the top shelf to his room answer question (II): “If not the older boy but rather the little girl had come, where would the sweets be?” by indicating the little girl’s room. This suggests that permanence should be understood in terms of generics, not of normal behaviour. But this has not been tested.

## 6. Conclusion

This paper isolated two differences between BCR and CFR. Faced with a counterfactual question about a story whose antecedent is inconsistent with the story, BC reasoners do not take nonpermanent features of the story into account. CF reasoners take all features of the story into account. As a result CF reasoners face a challenge that BC reasoners do not face in determining question worlds. CF reasoners must reorder the set of possible worlds weakly centered on their scenario worlds. BC reasoners, who find an antecedent world in the intersection of the set of propositions that they take into account, have no need to reorder. As a result the abilities required for BCR are a proper subset of the abilities required for CFR, which explains why CFR appears later in the developmental process. Finally, the possibility that children reason generically may answer open questions about children’s responses to counterfactual questions.

We used counterfactual logic to make our claims precise. We do not commit ourselves to possible worlds semantics for counterfactuals. Possible worlds semantics provides an effective means of spelling out the distinction between BCR and CFR, and has yielded testable predictions. But we do not have reasons to think the distinction could not be spelled out using other tools, possibly yielding different predictions. The choice of formalization would then be an empirical issue decided by which predictions are better supported by the evidence. For example, on a probabilistic semantics for counterfactuals along the lines of [5-7], when one affirms a counterfactual one endorses a high degree of confidence in the consequent under the supposition of the antecedent as, in one’s own view, the right one to have had at an earlier time. This later-time view may be informed by information gathered since that earlier time. As she writes, describing Adams’ view on counterfactuals, “the idea is that the (conditional) probability to be attached at the time of utterance to ‘If he had had the operation, he would have been cured’ is that which you now endorse for the (hypothetical) earlier indicative judgement, ‘If he has the operation, he will be cured”’ ([7], p. 4). For example, at an earlier time t_1_ , we may have had high confidence in the indicative conditional. Suppose that he decided not to have the operation and at a later time t_2_ it comes to light that he had a heart condition, and would likely have died (and so not been cured) if he had had the operation. Then at t_2_ it is right to have low confidence in ‘If he had had the operation, he would have been cured’. Further, from the perspective of t_2_ , our information about the heart condition gives us reason to think that it was right, at t_1_ , to have had low confidence in ‘If he has the operation, he will be cured’.

We do not know how Edgington would account for counterfactual questions posed about stories, but it might involve finding the appropriate confidence in the consequent given the antecedent in conjunction with the certain facts from the story. Then the difference between BCR and CFR might be spelled out in terms of abilities to determine what should be conjoined with the antecedent. We do not know if such an account can be developed, but we provide no reasons here why it could not.

## Footnotes

1 Some alternative explanations for why children fail to perform like adults on various counterfactual tasks are discussed in [1].

2 We do not wish or need to commit ourselves to any view of the process via which reasoners accomplish the formal goals we describe here. Some of the goals may be accomplishable by diverse processes.

3 We will here adopt Lewis’ variably strict semantics for counterfactuals, on which a counterfactual is interpreted relative to a similarity assignment $. $ assigns, to each possible world w, a similarity ordering: a set $_*w*_ of sets of worlds that meets these four conditions:

- $_*w*_ is nested: if S and T are members of $_*w*_, then either S ⊆ T or T ⊆ S.
- $_*w*_ is closed under unions: if S ⊆ $_*w*_, then ∪S ∈ $_*w*_.
- $_*w*_ is closed under nonempty intersections: if S ≠ Ø and S ⊆ $_*w*_, ∩S ∈ $_*w*_.
- (SC) $_*w*_ is strongly centered on w: the set {w} ∈ $_*w*_([13], pp. 13-14).

A counterfactual A□→C is nonvacuously true at w iff there is a S ∈ $_w_ that contains a world where A is true and A⊃C is true at every world in S ([13], p. 16). We treat (SC) as a default that may be overridden in some contexts; we describe one such context in this paper. In those contexts it is to be replaced with (WC). (WC) $_*w*_ is weakly centered on w: w belongs to every nonempty member of $_*w*_ and there is a nonempty member of $_*w*_ ([13], p. 29). When $_*w*_ is weakly centered and a set of worlds S = {w, w_1_, … w_*n*_ is the smallest member of $_*w*_, and $_*w*_ = $_*w*_1__ = … = $_*w_n_*_, we will denote this ordering $_*S*_. Throughout we adopt the limit assumption (that for any proposition p and ordering $_*w*_ there is a world that is maximally similar to w where p is true) because it lets us use less complicated language, though Lewis demonstrated that it is not necessary ([13], pp. 14 and 19-21).

4 Twenty-one adults (M = 30;2; SD = 6;6) were asked 4 different versions of question (I) out of the blue. They agreed on the answers in 96% of the questions (unpublished pilot data from Eva Rafetseder conducted at University of Salzburg).

5 The difference between weak and strong centering is described in ([13], p. 14, p. 29); see also footnote 2 herein. Intuitively, strong centering assumes that every world is more similar to itself than any other world, while weak centering allows that nonidentical worlds may be as similar to each other as one of them is to itself. We wish to capture the idea that no world where the experimenter’s story is true and that is maximally similar to the actual world is a better center for a set of spheres for evaluating a counterfactual question about the story than any other world where the experimenter’s story is true and that is maximally similar to the actual world.
